# Question order effects: how robust are survey measures on political solidarities with reference to Germany and Europe?

**DOI:** 10.1080/13645579.2023.2227011

**Published:** 2023-06-23

**Authors:** Jan Karem Höhne, Achim Goerres

**Affiliations:** a DZHW, University of Hannover, Hannover, Germany; b University of Duisburg-Essen, Duisburg, Germany

**Keywords:** Response behavior, data quality, non-probability panel, survey question order, web survey

## Abstract

The measurement of political solidarities and related concepts is an important endeavor in numerous scientific disciplines, such as political and social science research. European surveys, such as the Eurobarometer, frequently measure these concepts for people’s home country and Europe raising questions with respect to the order of precedence. Research has shown that the order of asking questions can have a profound impact on answer behavior compromising data quality. In this study, we therefore investigated the occurrence of question order effects in a German-European context using two questions on political solidarities. For this purpose, we conducted an experiment in a German online panel (*N* = 874) and analyzed response behavior and effort in terms of response times. In contrast to previous research, we found no empirical evidence for question order effects impacting people’s responses. Even though there were no response time differences between the question order conditions, the first question always took longer to respond to than the remaining one. Overall, our findings indicate the robustness of questions on political solidarities against question order effects. One potential explanation is that people have comparatively strong (or crystallized) attitudes when it comes to political solidarities.

## Introduction and research questions

Measuring political solidarities – i.e. the individual willingness to share costs by public redistribution favoring other people than oneself (Goerres & Höhne, 2023) – is key in political science research and many adjacent research fields to infer public opinion. Accordingly, many established social surveys, such as the Eurobarometer and European Social Survey (ESS), frequently employ survey questions on political solidarities and related concepts, such as redistribution and welfare chauvinism. In order to draw a comprehensive picture, these surveys commonly consider people’s attitudes towards their home country as well as towards Europe. However, as shown by Silber et al. (2016), people tend to provide more positive responses regarding their home country than Europe. Interestingly, most frequently, people are first asked about their home country and then about Europe. By experimentally varying the question order (Germany-Europe vs. Europe-Germany), Silber et al. (2016) found empirical evidence that the question order impacts response behavior. For example, the authors reported that people indicated more solidarity (or identification) with the European Union when this question was asked before the identical question with reference to Germany. Such measurement artefacts could skew the assessments in studies of European solidarity because of considerable disagreement about the level of the redistributive political system where solidarity may be strongest (Gerhards et al., 2019; Lahusen & Grasso, 2018).

Question order effects can be subsumed under the umbrella of context effects (Schwarz, 1991; Sudman et al., 1996). In the special case of a German-European comparison, it can be assumed that people understand both Germany and Europe as different entities. Especially since citizen relationships towards these two entities have been politicized by political actors since the 1990s (Hooghe & Marks, 2005), even though Germany falls into the geographic and political area of Europe. This can result in question order effects due to a so-called part–part comparison in which the first question sets a frame of reference or standard of comparison for the following question (Silber et al., 2016; Stefkovics & Kmetty, 2022; Tourangeau et al., 2000). In the case of a German-European question order, it is assumable that respondents compare Europe to Germany (their home country) when evaluating Europe. Having a German comparison standard in mind, this potentially impacts responding to the question on Europe. Following the findings by Silber et al. (2016) and our reasoning regarding a part–part comparison, it is to expect that respondents express fewer positive attitudes towards Europe when this question is asked second (after the question on Germany).

By unraveling the cognitive process of responding to survey questions in its main components (i.e. comprehension, retrieval, judgment, and response), question order effects can be associated with the information retrieval and judgement formation (Tourangeau et al., 2000) and occur when information and judgments of previous questions resonate with later questions (Stefkovics & Kmetty, 2022). This results in measurement error reducing data quality and weakens the significance of the conclusions that can be drawn from survey data.

Considering the survey literature, there are various empirical studies and scientific contributions on question order effects (see, for example, Schuman & Presser, 1981; Schwarz, 1991; Silber et al., 2016; Stark et al., 2020; Stefkovics & Kmetty, 2022; Sudman et al., 1996; Tourangeau et al., 2000). However, these studies mainly consider people’s response behavior in terms of response distributions. In contrast, studies considering the response effort associated with different question orders are very rare. Thus, the current state of research lacks important knowledge about the association between question order and response effort. Response effort is a key aspect in question and questionnaire design and crucial for understanding response behavior of respondents (Bradburn, 1978).

In this study, we contribute to the current state of research by investigating both response distributions and effort of different question orders. Specifically, response effort is evaluated in terms of response times (see Höhne et al. (2017) and Lenzner et al. (2010) for a similar strategy). For this purpose, we conducted an experiment in a German online panel and randomly assigned participants to one out of two question orders. In doing so, we attempt to answer the following two research questions:

1. How do different orders of questions on political solidarities in a German-European context impact response behavior in terms of response distributions?
2. How do different orders of questions on political solidarities in a German-European context impact response effort in terms of response times?

## Method

### Data

Data were collected in the non-probability SoSci Panel (www.soscipanel.de). The SoSci Panel is a project of the Institute for Communication Science and Media Research at the Ludwig-Maximilian-University Munich and the German Society for Journalism and Communication Science (DGPuK). It does not pursue any commercial goals. Researchers are eligible to submit study proposals that undergo a review process evaluating the methodological soundness of the studies. By proposal acceptance, respondents of the SoSci Panel (recruited via an opt-in subscription process) are invited to take part in the web surveys. The email invitation process is administered by the panel staff. Web survey data collection is free of charge.

The web survey ran from 16th May 2022 to 5th June 2022 (with a reminder sent on 25th May 2022). The invitation email was sent to 5,676 respondents (out of these email recipients, 68 could not be successfully reached). Invitations included information on the topic, the estimated duration of the web survey (approx. 20 minutes), and a link to the web survey. The first page of the web survey provided additional details on the web survey and its structure. We also included a statement of confidentiality, expounding that the study adheres to EU and national data protection laws and regulations. Respondents took part voluntarily without the provision of incentives. The questions of this study were placed in the last quarter of the web survey. For replication purposes, data and analysis code are available through Harvard Dataverse (see https://doi.org/10.7910/DVN/UKRKLU).

### Sample

In total, 874 respondents participated in this experimental study, which results in a participation rate of 15%. On average, these respondents were  49 years old, and 63% of them were female.^1^ In terms of education, 5% had completed lower secondary school or less (low education level), 15% intermediate secondary school (medium education level), and 79% college preparatory secondary school or university-level education (high education level).

### Experimental design

Respondents were randomly assigned to one of the two experimental groups. The first group (*n* = 427) received first the question on Germany and then the question on Europe (*Germany-Europe condition*). The second group (*n* = 447) received first the question on Europe and then the question on Germany (*Europe-Germany condition*). Evaluating the effectiveness of random assignment resulted in no differences between the two experimental groups with respect to age, gender, and education.

### Questions

Overall, we employed the following two questions on political solidarities with different reference points:

1) Please tell us to what extent do you agree with the following statement?

I am willing to endorse governmental support from the federal government for other people in Germany, even if I do not benefit from it.

2) Please tell us to what extent do you agree with the following statement?

I am willing to endorse governmental support by the European Union for other people in the EU, even if I do not benefit from it.

Depending on the experimental condition, the firstly asked question included a short introductory text: ‘Governmental support for others can include money, services, or favorable rules.’ We presented the questions on two web survey pages (single-question presentation) with 5-point, end labeled, vertically aligned rating scales running from ‘agree strongly’ to ‘agree not at all’.

## Results

### Research question 1

With respect to our first research question, we investigated whether and to what extent different question orders (Germany-Europe vs. Europe-Germany) resulted in different response distributions (scale ran from 1 ‘agree strongly’ to 5 ‘agree not at all’):

Condition 1 (Germany-Europe)

Germany 1st: 60.9%, 22.4%, 11.6%, 2.4%, and 2.8%

Europe 2nd: 55.7%, 22.0%, 14.7%, 3.8%, and 3.8%

Condition 2 (Europe-Germany)

Europe 1st: 51.3%, 27.2%, 14.7%, 3.2%, and 3.7%

Germany 2nd: 56.7%, 25.7%, 14.0%, 1.8%, and 1.8%

The inspection of the response distributions shows that they are skewed. However, the order of the questions did not substantially impact people’s response behavior. There is no higher willingness (in the form of agreeing answers) to endorse governmental support depending on the question order. This impression was supported by the results of two chi-square tests: Germany [χ^2^ = 3.80, df = 4, p-value = 0.43] and Europe [χ^2^ = 3.36, df = 4, p-value = 0.50]. Both tests indicated no statically significant differences.

### Research question 2

With respect to our second research question, we investigated whether and to what extent different question orders resulted in different response efforts. Following Höhne et al. (2017) and Lenzner et al. (2010), we used response times as an indicator of the effort required to understand and respond to the questions on political solidarities in a German-European context. Responding to the first of the two questions took longer than responding to the second one.

Condition 1 (Germany-Europe)

Germany 1st: 16 seconds

Europe 2nd: 8 seconds

Condition 2 (Europe-Germany)

Europe 1st: 16 seconds

Germany 2nd: 9 seconds

The overall median response times of the question order conditions did not differ. This impression was supported by the result of a non-parametric median test: [χ^2^ = 1.38, df = 1, p-value = 0.24]. The test result indicated no statically significant difference.

## Discussion and conclusion

The aim of this study was to investigate whether and to what extent people’s responses to questions on political solidarities in a German-European context are prone to question order effects. To this end, we conducted an experiment in a German online panel and randomly assigned people to one out of two question order conditions: Germany-Europe or Europe-Germany. The overall results indicated that effects of the question order are a minor threat to response behavior and effort when asking questions on political solidarities.

With respect to our first research question on response behavior, we found no differences in response distributions due to question order. In contrast to our expectation, respondents’ willingness to endorse governmental support by the EU for other people in the EU was not decreased when the Europe question was asked second (after the question on Germany). This finding indicates that people seemingly have strong attitudes on political solidarities. To put it differently, response effects, such as those of the question order, may be a consequence of poorly crystallized attitudes (Cantrill, 1944). This is only an attempted explanation for our findings that lacks empirical evidence. We therefore suggest that future research, for example, employs additional questions for evaluating people’s attitude strength or topic closeness.

With respect to our second research question on response effort we collected response times in addition to people’s responses. The response time analyses resulted in almost no differences indicating that altered question orders do not affect response effort. Interestingly, it took people consistently longer to respond to the first of the two questions. Although this may be partially due to the short introductory text that accompanied the first question (see sub-section ‘Questions’) and the fact that respondents become faster when receiving the same question with a different subject, it nevertheless indicates that previous questions serve as a frame of reference in which later questions are processed. However, in order to draw more robust conclusions about this claim, we encourage further studies that implement a more refined and elaborated question design strategy.

This study has some limitations that represent perspectives for future research. First, our study was conducted in one single country restricting our conclusions to Germany. We therefore suggest to go beyond Germany and to consider a cross-national comparison. Second, we conducted our study in a non-probability online panel with a highly educated sample. This may have impacted the empirical findings related to our two research questions. It would be worthwhile if future studies build on more advanced samples, such as from probability-based online panels. Finally, in this study, we only employed two questions on political solidarities that concerned redistribution. However, political solidarities are a multi-dimensional concept (Goerres & Höhne, 2023) and thus we recommend to consider a more diverse set of questions.

Irrespective of its limitations, this study contributes to the current state of research. Importantly, it reveals that question order effects are no major concern for response behavior and effort when it comes to measuring political solidarities in a German-European context. We take this as good news for past and future studies. Nonetheless, we advocate for being cautious during question and questionnaire design.

## Note

1. Approx. 2% (*n* = 15) of the respondents selected the response option ‘divers.’ The remaining respondents selected the response option ‘male.’
